# Characterization of a highly stable α-galactosidase from thermophilic *Rasamsonia emersonii* heterologously expressed in a modified *Pichia pastoris* expression system

**DOI:** 10.1186/s12934-019-1234-6

**Published:** 2019-10-23

**Authors:** Jian-Lu An, Wei-Xin Zhang, Wei-Ping Wu, Guan-Jun Chen, Wei-Feng Liu

**Affiliations:** State Key Laboratory of Microbial Technology, Shandong University, No. 72 Binhai Road, Qingdao, 266237 People’s Republic of China

**Keywords:** α-Galactosidase, *Rasamsonia emersonii*, Structural stability, Thermostability, *Pichia pastoris AOX1* promoter

## Abstract

**Background:**

Structurally stable α-galactosidases are of great interest for various biotechnological applications. More thermophilic α-galactosidases with high activity and structural stability have therefore to be mined and characterized. On the other hand, few studies have been performed to prominently enhance the *AOX1* promoter activity in the commonly used *Pichia pastoris* system, in which production of some heterologous proteins are insufficient for further study.

**Results:**

*ReGal2* encoding a thermoactive α-galactosidase was identified from the thermophilic (hemi)cellulolytic fungus *Rasamsonia emersonii*. Significantly increased production of ReGal2 was achieved when *ReGal2* was expressed in an engineered *Pastoris pichia* expression system with a modified *AOX1* promoter and simultaneous fortified expression of Mxr1 that is involved in transcriptionally activating *AOX1*. Purified ReGal2 exists as an oligomer and has remarkable thermo-activity and thermo-tolerance, exhibiting maximum activity of 935 U/mg towards *p*NPGal at 80 °C and retaining full activity after incubation at 70 °C for 60 h. ReGal2 is insensitive to treatments by many metal ions and exhibits superior tolerance to protein denaturants. Moreover, ReGal2 efficiently hydrolyzed stachyose and raffinose in soybeans at 70 °C in 3 h and 24 h, respectively.

**Conclusion:**

A modified *P. pichia* expression system with significantly enhanced *AOX1* promoter activity has been established, in which ReGal2 production is markedly elevated to facilitate downstream purification and characterization. Purified ReGal2 exhibited prominent features in thermostability, catalytic activity, and resistance to protein denaturants. ReGal2 thus holds great potential in relevant biotechnological applications.

## Introduction

In addition to glycoproteins and glycolipids, α-linked galactosyl residues are found in nature in two major forms: (1) as branched residues in galactomannans and galactoglucomannans that are classified into hemicellulose [1, 2], the second most abundant biopolymer on earth after cellulose, both of which are mainly present in plant cell walls, and (2) as moieties of oligosaccharides including melibiose, raffinose, and stachyose that are present in sugar beets and soys [3].

α-Galactosidases (α-d-galactoside galactohydrolase; EC 3.2.1.22) are exo-glycosidases that catalyze the removal of α-linked terminal non-reducing galactose residues from different galactoside-containing polymers or oligopolysaccharides [3]. They are widely distributed in microorganisms, plants, and animals, and are classified into glycoside hydrolase (GH) families 4, 27, 36, 57, 97, and 110 [4]. Whereas most bacterial α-galactosidases fall into GH family 36, most fungal α-galactosidases belong to GH27.

α-Galactosidases are of great interest in a variety of biotechnological applications [1, 3]. In pulp and paper manufacturing, α-galactosidases are used with other kinds of hemicellulases to enhance pulp bleaching [1, 5]. These enzymes are also conventionally applied in food and feed industries to process soy molasses and soybean milk to remove anti-nutritional factors such as raffinose family oligosaccharides (RFOs) that are mainly raffinose and stachyose [6], and to eliminate raffinose from sugar beet molasses to increase sucrose crystallization and consequently improve the yield [7].

Thermostable enzymes are frequently courted especially for processes running at elevated temperatures to achieve an overall high productivity [8]. High structural stability is also an important and desirable feature of enzymes to resist toxic inhibitors either derived from the substrate or generated during the reaction. Compared to extensive studies on the mesophilic α-galactosidases, only a few thermophilic/thermostolerant α-galactosidases have been characterized, most of which are from thermophilic fungi (see a summary in Table 1), *e.g*., *Neosarotrya fischeri* [9, 10], *Thermomyces lanuginosus* [11], *Talaromyces leycettanus* [12], and *Talaromyces emersonii* [13]. These α-galactosidases exhibit various degrees of thermo-activity and thermo-stability as well as resistance to a number of metal ions and some other chemicals (see a summary in Table 5). However, none has been found to be tolerant to protein denaturants such as SDS, urea, and guanidine hydrochloride (GdhHCl), implicating their structural fragility to these denaturants. Therefore, more thermophilic/thermotolerant α-galactosidases with desired properties including high structural stabilities have to be mined and characterized.

**Table 1:** Comparison of enzymatic properties of ReGal2 with other thermophilic fungal GH27 α-galactosidases

Organism	Optimal pH	Optimal temperature/thermostability	*K*_m_ (mM)	*V*_max_ (μmol min^−1^ mg^−1^)	*k*_cat_ (s^−1^)	*K*_cat_/*K*_m_ (s^−1^mM^−1^)	References
*Talaromyces emersonii*	4.0	80 °C/70 °C for 60 h	0.19	566.5	623.15	3279.7	This study
*T. leycettanus JCM12802*	4.0	70 °C/60% at 65 °C for 1 h	1.32	389.8	341	258.4	[12]
*Neosartorya fischeri P1*	4.0	75 °C/60 °C for 1 h	2.84	1850	1621	570.8	[10]
*Talaromyces emersonii*	4.5	70 °C/50 °C for 10 days	0.29	240.3	200.3	690.5	[13]
*Candida javanica*	4.0	70 °C/70% at 70 °C for 15 min	11	100	–	–	[36]
*Thermomyces lanuginosus*	4.5–5.0	65–70 °C/60 °C for 6 h	0.5	52.4	49.8	97.8	[11]
*Aspergillus terreus*	5.0	65 °C/65 °C for 30 min	0.11	7.2	–	–	[37]
*Neosartorya fischeri P1*	4.5	60 °C/60 °C for 10 min	0.8	449.5	368.6	460.8	[9]
*Penicillium canescens*	4.0–5.0	55 °C/40–50 °C for 3 h	0.48	6.89	7	14.6	[38]
*Agaricus bisporus*	4.0	60 °C/–	0.30	193.12	–	–	[39]
*Rhizomucor miehei*	4.5	60 °C/55 °C for 30 min	0.36	378.9	6.32	17.5	[32]
*Irpex lacteus*	4.8	70 °C/90% at 60 °C for 10 h	1.2	1012	–	1900	[40]

*Rasamsonia emersonii* (former name *Talaromyces emersonii*) is a (hemi)cellulolytic filamentous fungus with an optimum growth temperature of 40–45 °C and can even grow at up to 55 °C [14]. Since its isolation, *R. emersonii* has become as a rich source of thermophilic polymer-degrading enzymes, *e.g.*, β-glucosidase, endo-1,4-β-glucanase, and xylanase [15–17]. In particular, an α-galactosidase from this fungus exhibits remarkable thermostability and specific activities, which holds great potential for industrial application [13].

The methylotrophic yeast *Pichia pastoris* is one of the most commonly used expression systems for heterologous protein production, especially for eukaryotic proteins [18–20]. The highly inducible and tightly-regulated alcohol oxidase 1 gene (*AOX1*) promoter is extensively used to achieve high-level heterologous protein expression under methanol condition [18]. In recent years, several strategies via engineering transcription factors exerting regulatory effects on the *AOX1* promoter, including overexpression of the activators Mxr1 and Mit1 [21–24] and/or elimination of the repressors Mig1 and Mig2 [23], have been adopted to achieve the activation of the *AOX1* promoter under non-inducing conditions (*e.g.*, glucose and glycerol). Nevertheless, few studies have been performed to prominently enhance the *AOX1* promoter activity on methanol, under which condition the production of some heterologous proteins is still too low to obtain decent amounts for further study.

In this study, four putative α-galactosidase encoding genes from *R. emersonii* were cloned and expressed in *P. pastoris*. ReGal2 displaying the highest optimum temperature was selected for further characterization. ReGal2 expression was significantly enhanced in an engineered *P. pastoris* expression system, wherein overexpression of the transcriptional activator Mxr1 was combined with the expansion of the corresponding *cis*-elements within the *AOX1* promoter. Purified ReGal2 exhibited high hydrolytic activity as well as pronounced thermostability and tolerance to protein denaturants compared to the already reported fungal counterparts.

## Materials

### Strains and cultivation conditions

*Rasamsonia emersonii* was cultured on potato dextrose agar (PDA) plates at 45 °C. *P. pastoris* GS115 was cultured on yeast extract peptone dextrose (YPD) medium at 30 °C. *Escherichia coli* DH5α was routinely cultured at 37 °C on Luria–Bertani (LB) broth.

### Heterologous expression of *R. emersonii* α-galactosidases in *P. pastoris* GS115

*Rasamsonia emersonii* was cultured in liquid potato dextrose medium at 45 °C for 2 days, and its mycelia was collected by filtration, and subsequently applied for extraction of genomic DNA using the E.Z.N.A. Fungal DNA Mini kit (Omega). The full-length coding sequences of *ReGal2*–*ReGal4*, but without introns and the corresponding sequences encoding N-terminal signal peptides that were predicted by SignalP (http://www.cbs.dtu.dk/services/SignalP/), were amplified from the *R. emersonii* genomic DNA, inserted into the pPICZαA vector (Invitrogen), and transformed into *P. pastoris* GS115, respectively. The heterologous expression of *ReGal5* with removal of introns and the coding sequence for N-terminal transmembrane region predicted by TMHMM (http://www.cbs.dtu.dk/services/TMHMM/) was performed using the same strategy. Transformation of *P. pastoris* using electroporation was carried out according to the *Pichia* expression system manual (Invitrogen). The correct integration events occurred in the transformants were verified by anchored PCR. The crude culture supernatant of the correct transformants with 0.5% methanol induction was collected and subjected to enzymatic activity and SDS-PAGE analyses.

### Engineering of *P. pastoris* GS115 to enhance *AOX1* promoter activity

The transcriptional activators Mxr1 and Mit1 encoding genes were respectively overexpressed in *P. pastoris* GS115 under the control of the constitutive *GAP* promoter [25]. Specifically, the amplified *GAP* promoter was inserted into the pPICZαA plasmid that was digested with *Bgl*II and *Kpn*I to remove the original *AOX1* promoter, generating pPICZαA-*GAP*. The full-length coding sequence of *Mxr1* or *Mit1* was inserted into the *Kpn*I/*Xba*I-digested pPICZαA-*GAP* to construct pPICZαA-*GAP*-*Mxr1* and pPICZαA-*GAP*-*Mit1*, respectively. To increase the activator response elements within the *AOX1* promoter, the − 719 to − 503 promoter fragment upstream the initiation ATG codon, was amplified and fused to the 5′ end of the native *AOX1* promoter (− 1 to − 936) via overlap extension PCR [26], generating the *MBS*-*AOX1* promoter. To evaluate the activities of the native or engineered *AOX1* promoter in *P. pastoris*, the enhanced green fluorescence protein encoding gene (*egfp*) was fused to the 3′ end of the promoters. Specifically, the full-length *egfp* fragment was inserted into the *Eco*RI-digested pPIC3.5 k*, which was derived from pPIC3.5 k (Invitrogen) by eliminating the *Bgl*II at the +6603 site and therefore retaining only one *Bgl*II at the +2 site. The resultant plasmid, pPIC3.5 k*-*egfp*, carrying the *egfp* gene readily behind the native *AOX1* promoter, was transformed into *P. pastoris* GS115 cells, to result in the control-*P*_*AOX1*_ transformants. The plasmids pPICZαA-*GAP*-*Mxr1* and pPICZαA-*GAP*-*Mit1* were linearized with *Avr*II and transformed into the control-*P*_*AOX1*_ cells to result in the OE*Mxr1* and OE*Mit1* transformants, respectively. Moreover, the *MBS*-*AOX1* promoter fragment was inserted into the plasmid pPIC3.5 k*-*egfp* that was simultaneously digested with *Bgl*II and *Eco*RI, to replace the native *AOX1* promoter. The resultant plasmid was linearized with *Mss*I and transformed into *P. pastoris* GS115 to generate the *P*_*MBS*-*AOX1*_ transformants. The plasmid pPICZαA-*GAP*-*Mxr1* was further transformed into *P*_*MBS*-*AOX1*_ to generate the OE*Mxr1*+*P*_*MBS*-*AOX1*_ transformants.

### Fluorometric analysis

Quantification of fluorescence intensity from GFP was detected using a Nikon Eclipse 80i fluorescence microscope. *P. pastoris* transformants were cultured with 1% glycerol and then equally transferred to medium containing 0.5% methanol. Cells grown to late exponential phase were harvested and transferred to a black microtiter plate and measured using a 96-well spectrofluorometer at an excitation wavelength of 485 nm and an emission wavelength of 535 nm. An aliquot of each sample was diluted for OD_600_ determination, to calculate the relative fluorescence for each sample per OD.

### Expression and purification of ReGal2 in the engineered *P. pastoris* expression system

#### Expression of ReGal2 in the engineered expression system

To compare ReGal2 production in the conventional and engineered *P. pastoris* expression systems, the α-factor signal sequence encoding sequence was amplified from the pPIC9 k vector (Invitrogen) and fused to the 5′ end of the coding sequence of *ReGal2* with removal of introns and the first 69 bp encoding the putative N-terminal signal peptide. The resultant fragment was ligated into the *Eco*RI-digested pPIC3.5 k*, generating the pPIC3.5 k*-*ReGal2* plasmid, which was transformed into *P. pastoris* GS115 to generate the control-*ReGal2* transformants. Moreover, to drive the expression of *ReGal2* via the *MBS*-*AOX1* promoter, the − 936 to − 730 fragment of the native *AOX1* promoter within pPIC3.5 k*-*ReGal2* was replaced by the − 1152 to − 730 fragment from the *MBS*-*AOX1* promoter via *Bgl*II/*Sac*I-mediated ligation. The resultant plasmid and the pPICZαA-*GAP*-*Mxr1* plasmid were then successively transformed into *P. pastoris* GS115.

#### Purification of ReGal2 in the engineered expression system

The crude culture supernatant of *P. pastoris* transformants with 0.5% methanol induction was fractioned through precipitation with ammonium sulfate ((NH_4_)_2_SO_4_). Briefly, 55.9 g of ground (NH_4_)_2_SO_4_ was added to 100 mL of fermentation broth. The mixture was maintained at 4 °C overnight and centrifuged at 10,000 rpm for 20 min. The supernatant was discarded and the pellet was resuspended with twofold volumes of 10 mM Tris–HCl buffer (pH 8.0) containing 100 mM NaCl. The mixture was dialyzed to remove (NH_4_)_2_SO_4_, and subjected to size exclusion chromatography using the ÄKTA™ system (GE Healthcare) equipped with a column of Superdex™ 200 Increase 10/300 Gl. Chromatography was performed using 10 mM Tris–HCl buffer (pH 8.0) plus 100 mM NaCl. Fractions were collected and subjected to enzymatic activity and SDS-PAGE analyses.

### Enzymatic characterization

#### α-Galactosidase activity assays

The α-galactosidase activity was determined using the *p*-nitrophenyl-α-d-galactopyranoside (*p*NPGal) as substrate. The assays were performed in 200 μl of reaction mixtures containing 10 μl of diluted culture supernatant or purified enzyme and 190 μl *p*NPGal dissolved in 20 mM HAC-NaAC buffer (pH 4.8). The reaction mixture was then incubated at 50 °C for 10 min. The reaction was terminated by adding 50 μl of 10% Na_2_CO_3_. One unit of the enzyme activity (IU) was defined as the amount of enzyme releasing 1 μmol of *p*NP per minute. The kinetic parameters were determined at 80 °C using different *p*NPGal concentration (ranging from 0.25 to 2.0 mM). The Michaelis constant (*K*_*m*_) and *V*_*max*_ were calculated by non-linear regression analysis using Origin 8.5 Pro software.

#### Protein assays

Purified protein concentration was determined using a Pierce™ BCA Protein Assay kit with standard bovine serum albumin (BSA) as the standard protein.

#### SDS-PAGE and semi-native PAGE

SDS-PAGE analyses were performed essentially as previously described [27], except for a modification in sample preheating time that was extended to 15 min. To analyze the polymeric state of ReGal2, samples treated with SDS and β-mercaptoethanol, but without 15-min boiling, were subjected to SDS-PAGE. To analyze the hydrolytic activity against methylumbelliferyl-α-d-galactopyranoside (MUG), ReGal2 with or without heating treatment was loaded to the polyacrylamide gel containing 25 μg ml^−1^ of MUG. After electrophoresis, the gel was immersed in 20 mM HAC-NaAC buffer at pH 4.8 and incubated for 30 min. The release of methylumbelliferone was visualized under ultraviolet light.

#### Molecular weight determination

The molecular weight of ReGal2 was determined using size exclusion chromatography with standards (GE Healthcare) as follows: ovalbumin (43 kDa), conalbumin (75 kDa), aldolase (158 kDa), ferritin (44 kDa) and thyroglobulin (669 kDa).

#### Determination of optimal temperature and pH as well as thermostability

The optimum temperature of the crude culture supernatant or purified ReGal2 was determined by measuring the hydrolytic activity toward *p*NPGal in 20 mM HAC-NaAC buffer (pH 4.8) at a temperature range from 40 to 100 °C. The thermostability of ReGal2 was determined by measuring the residual enzyme activity after incubation of the enzyme at different temperatures for the indicated time period. The optimum pH of purified ReGal2 was determined by measuring its hydrolytic activity toward *p*NPGal in different buffers of pH 2–10 at 50 °C. The Na_2_HPO_4_-citric acid and Tris–HCl buffers were used for pH 2.0–8.0 and pH 8.0–9.0, respectively.

#### Effect of the addition of metal ions, protein denaturants and other chemicals

The effect of metal ions on ReGal2 activity was determined by measuring the residual enzymatic activity at 50 °C in the presence of 0–20 mM of NiSO_4_, KCl, MnSO_4_, CuSO_4_, CoCl_2_, ZnSO_4_, or AgNO_3_. The effect of 0–3 M NaCl on ReGal2 activity was determined similarly. The effect of protein denaturants on ReGal2 activity was assessed by measuring the residual enzymatic activities at 50 °C in the presence of 0–200 mM of SDS, Urea, guanidine hydrochloride (GdnHCl), β-mercaptoethanol or EDTA.

#### Differential scanning calorimetry (DSC)

Thermal unfolding of ReGal2 was determined on a MicroCal VP-Capillary DSC calorimeter (Malvern, UK) with a range of 20–110 °C at a scan rate of 90 °C h^−1^. The protein concentration was 0.5 mg ml^−1^.

### Soybean treatment with ReGal2

One gram of defatted and ground soybean flour was suspended in 4 ml of 20 mM HAC-NaAC buffer (pH 4.8), shaken for 10 min, and centrifuged at 10,000 rpm for 20 min. After removal of undissolved residues, 300 μl of soymilk was treated with 100 μl of 2 U ml^−1^ ReGal2 at 50 °C or 70 °C for continuous time periods. The mixture was boiled for 15 min to terminate the reaction, followed by centrifugation at 10,000 rpm for 10 min. The resultant supernatant was subjected to high-performance liquid chromatography (HPLC) analysis that was performed on a REZEX ROA (0138) column with a mobile phase of 0.5632:1000 (v/v) H_2_SO_4_:H_2_O at a flow rate of 0.5 ml min^−1^ and an injection volume of 10 μl.

### Sequence analysis

Amino acid sequences of α-galactosidases were retrieved from the NCBI database (https://www.ncbi.nlm.nih.gov/). Protein domain organization was analyzed on NCBI. The phylogenetic tree was generated with MEGA 7 [28].

## Results and discussion

### Identification of a thermophilic α-galactosidase ReGal2 from the thermophilic fungus *R. emersonii*

**Fig. 1:**
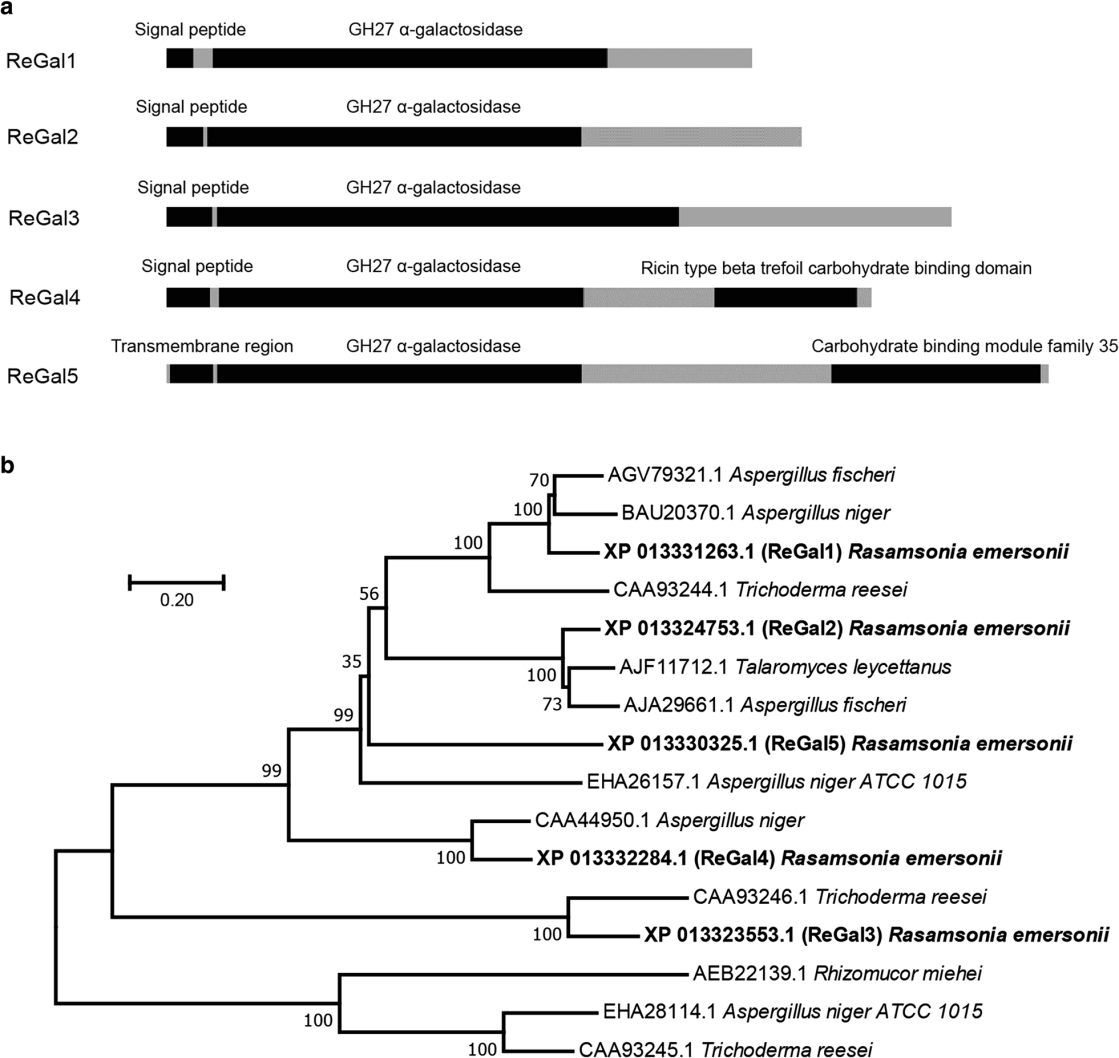
**a** Schematic illustration of domain organization of the α-galactosidases ReGal1–ReGal5 and **b** phylogenetic analysis of ReGal1–ReGal5 and their homologues from other filamentous fungi. ReGal1–ReGal5 were represented in bold. Amino acid sequence alignment was performed using Clustal W [35]. Phylogenetic analysis was performed with MEGA 7 [28] using neighbor-joining method with 1000 bootstraps

In order to mine α-galactosidases with ideal properties, the genome of the thermophilic (hemi)cellulolytic fungus *R. emersonii* were analyzed, and five putative α-galactosidase-encoding genes (*ReGal1–ReGal5*) were retrieved. The five corresponding proteins, ReGal1–ReGal5, are predicted to have a catalytic domain of GH27 family although their overall sequence identify is relatively low (< 34%; Fig. 1). All proteins except ReGal5 have putative signal peptides, suggesting that these enzymes may be secreted extracellularly. ReGal5 instead contains a putative N-terminal transmembrane region and was therefore predicted to be anchored to cell membrane in *R. emersonii*.

**Fig. 2:**
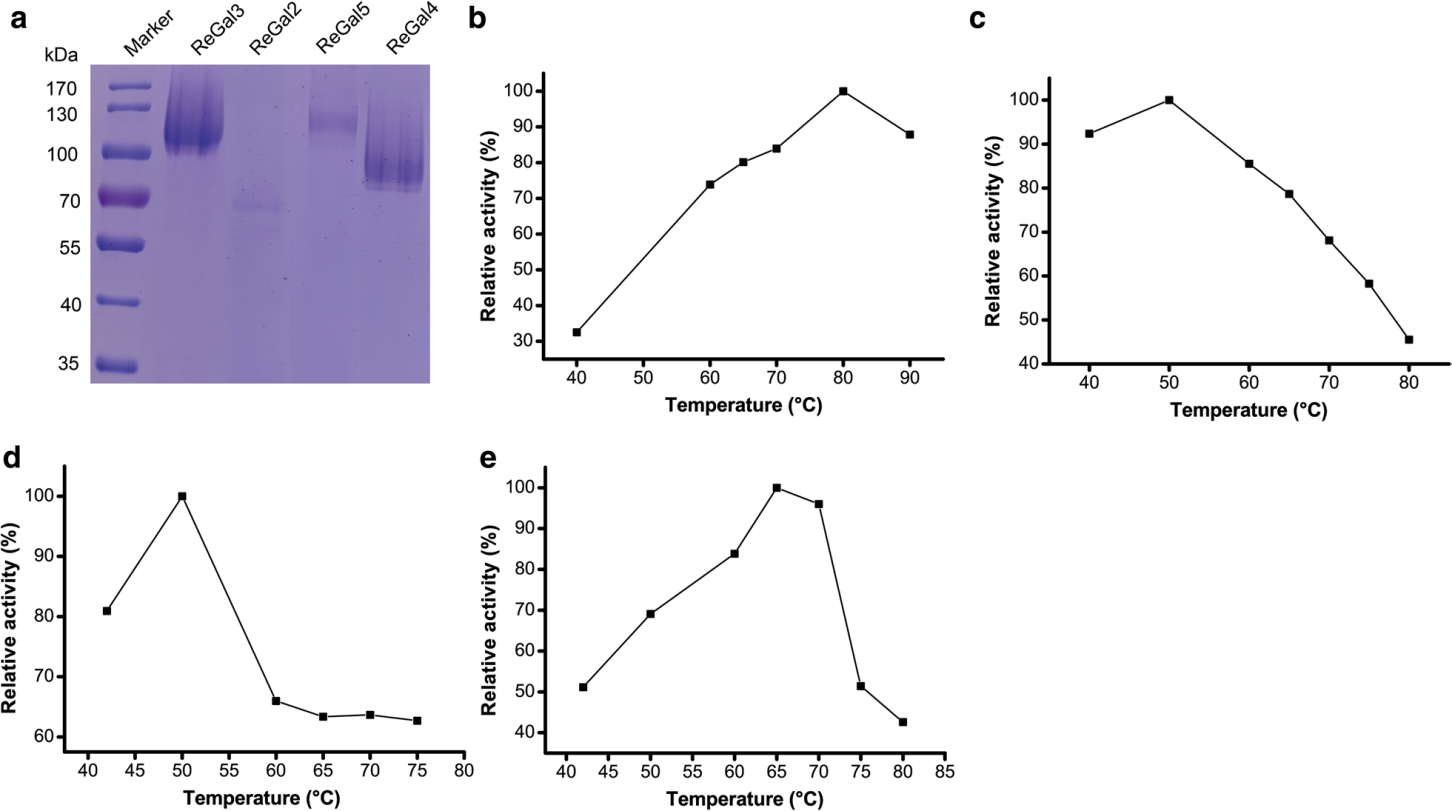
Heterologous production of ReGal2 to ReGal5 in *P. pastoris* GS115 and determination of their optimum temperatures. **a** SDS-PAGE analysis of the crude culture supernatant of *P. pastoris* GS115 transformants expressing ReGal2 to ReGal5, respectively. **b**–**e** Determination of the optimum temperatures of ReGal2 (**b**), ReGal3 (**c**), ReGal4 (**d**), and ReGal5 (**e**) with the crude culture supernatant of respective *P. pastoris* transformants after 0.5% methanol induction

Of the five *ReGal* genes, *ReGal1* has been previously reported to encode a thermophilic α-galactosidase [13]. We therefore performed the heterologous expression of *ReGal2*–*ReGal5*. These four genes without coding sequences for signal peptide or N-terminal transmembrane helix were individually expressed under the control of the *AOX1* promoter with methanol induction in *P. pastoris*. The resulting recombinant proteins all exhibited α-galactosidase activities as demonstrated by the remarkable hydrolytic activities toward *p*NPGal in the culture supernatant of the respective *P. pastoris* transformants (Table 2). However, SDS-PAGE analysis showed that the extracellular amounts of these recombinant α-galactosidases varied. The lowest production was observed for ReGal2 with barely detectable protein band on SDS-PAGE (Fig. 2a).

**Table 2:** Optimum temperatures of heterologously expressed ReGal2–ReGal5

Name	NCBI protein accession no.	Length (amino acids)	GH family	Optimum temperature (°C)	*p*NPGal activity (U ml^−1^)
ReGal2	XP_013324753.1	491	27	80	38.0
ReGal3	XP_013323553.1	607	27	50	20.7
ReGal4	XP_013332284.1	545	27	50	11.2
ReGal5	XP_013330325.1	682	27	65	3.5

The *p*NPGal hydrolytic activities and the optimal temperatures were determined with crude culture supernatant of *P. pastoris* transformants

The optimum temperature of each recombinant α-galactosidase was measured with the crude culture supernatant. As shown in Fig. 2 and Table 2, while ReGal3 and ReGal4 showed their maximum activity at 50 °C, ReGal2 and ReGal5 exhibited their highest activity at 80 °C and 65 °C, respectively, suggesting that ReGal2 and ReGal5 are potential thermophilic enzymes. The observation that these enzymes showed varying optimum temperatures may implicate a cooperative division of action among them, allowing the fungus to achieve the efficient degradation when confronted with changing environmental conditions. Given that ReGal2 is the most thermoactive enzyme among these α-galactosidases, we next focused on the purification and characterization of ReGal2.

### Improved expression and purification of recombinant ReGal2 in an optimized *P. pastoris* expression system with enhanced *AOX1* promoter activity

**Fig. 3:**
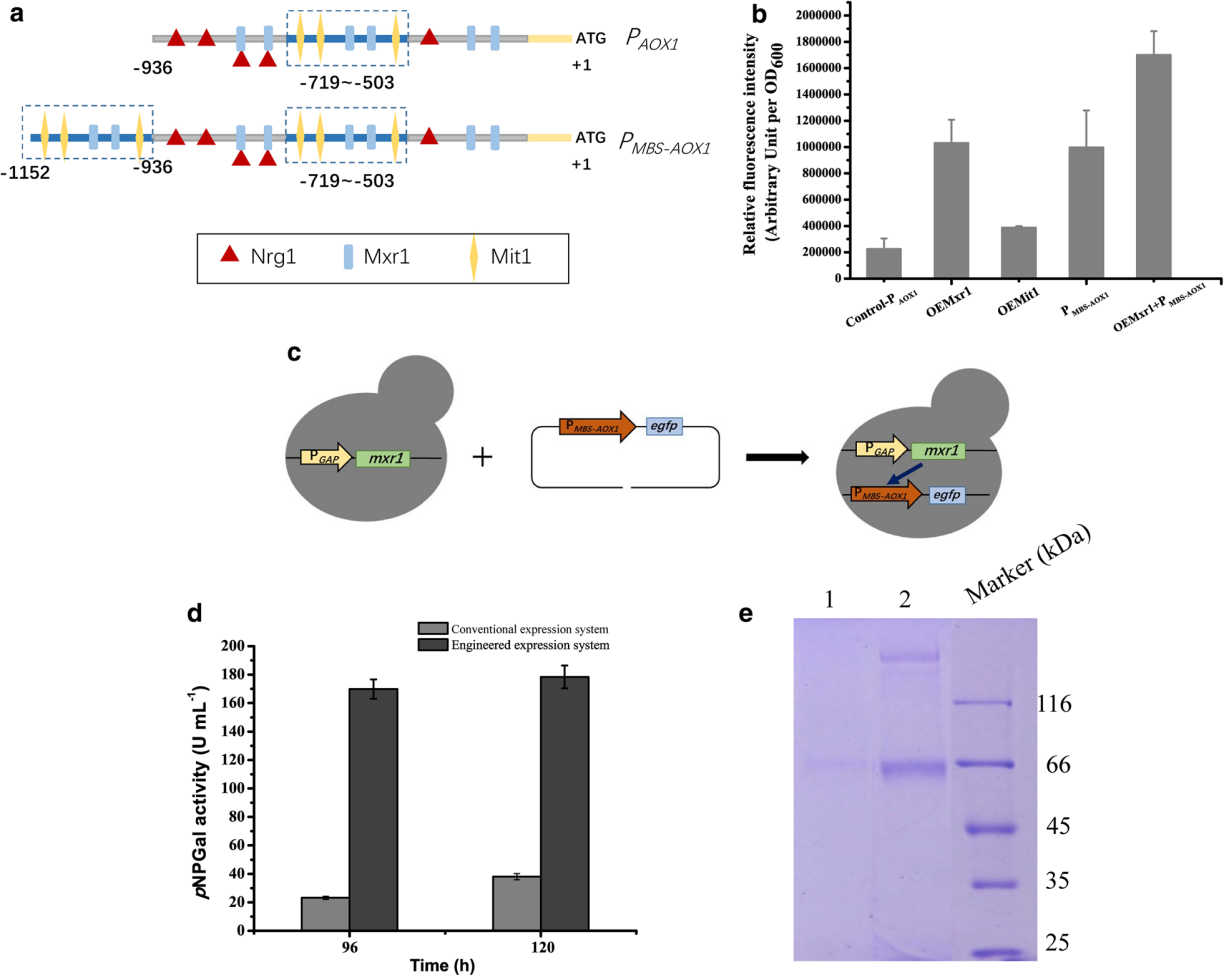
Markedly increased ReGal2 production in the optimized *P. pastoris* expression system with enhanced *AOX1* promoter activity. **a** Schematic illustration of the relevant *cis*-elements within the native *AOX1* promoter and the engineered *MBS*-*AOX1* promoter. **b** Evaluation of the *AOX1* promoter activity in the conventional or engineered *P. pastoris* expression system by determining the fluorescence intensity from the EGFP reporter protein. **c** Schematic illustration of the construction of the optimum engineered *P. pastoris* expression system that contains both *MBS*-*AOX1* promoter and overexpressed Mxr1. **d** Extracellular *p*NPGal hydrolytic activity of the culture supernatant from the conventional and the engineered *P. pastoris* cells expressing ReGal2, respectively. **e** SDS-PAGE analysis of the 120 h-culture supernatant of the conventional (lane 1) and the engineered *P. pastoris* cells (lane 2) expressing ReGal2, respectively. Samples was pretreated with SDS, β-mercaptoethanol and 5-min boiling according to standard protocols before being loaded for SDS-PAGE analysis

As shown above, the production yield of ReGal2 in *P. pastoris* is too low to obtain decent amounts of purified proteins for further characterization. Attempts were therefore made to improve protein production by enhancing the *AOX1* promoter activity via 1) overexpressing the transcriptional activators, Mxr1 and Mit1 [21, 22], and 2) expanding their putative binding elements within the *AOX1* promoter [22, 29] (Fig. 3a). The reporter gene *gfp* was fused to the native or the engineered *AOX1* promoter for evaluation of their activities via fluorescence intensity analysis. As shown in Fig. 3b, overexpression of Mxr1 or Mit1 driven by the constitutive *GAP* promoter markedly enhanced the *AOX1* promoter activity on methanol, with a more pronounced enhancement (4.5-fold in fluorescence intensity) resulted from Mxr1 expression. To make the most of the overexpressed Mxr1 or Mit1, we purposedly placed the − 719 to − 503 promoter region that contains putative binding motifs of Mxr1 or Mit1, but without the binding motif of the transcription repressor PpNrg1 [30], at the 5′ end of the *AOX1* promoter. This modified promoter resulted in a 4.4-fold increase in the fluorescence intensity compared to that driven by the native promoter (Fig. 3b). Combining these two strategies to further elevate the engineered *AOX1* promoter activity resulted in an overall 7.5-fold increase in the fluorescence intensity, compared to that in the conventional *P. pastoris* system (Fig. 3b, c).

*ReGal2* was then expressed in the optimized *P. pastoris* expression system. Compared with the conventional system, ReGal2 production was markedly enhanced, as shown by the significantly increased extracellular α-galactosidase activity and the readily detected protein band resolved by SDS-PAGE (Fig. 3d, e). The enhanced ReGal2 production facilitated the following two-step purification via ammonium sulfate precipitation and size-exclusion chromatography, with a final yield of ~ 15 mg l^−1^.

### Biochemical characteristics of the recombinant ReGal2

#### ReGal2 is an oligomer

**Fig. 4:**
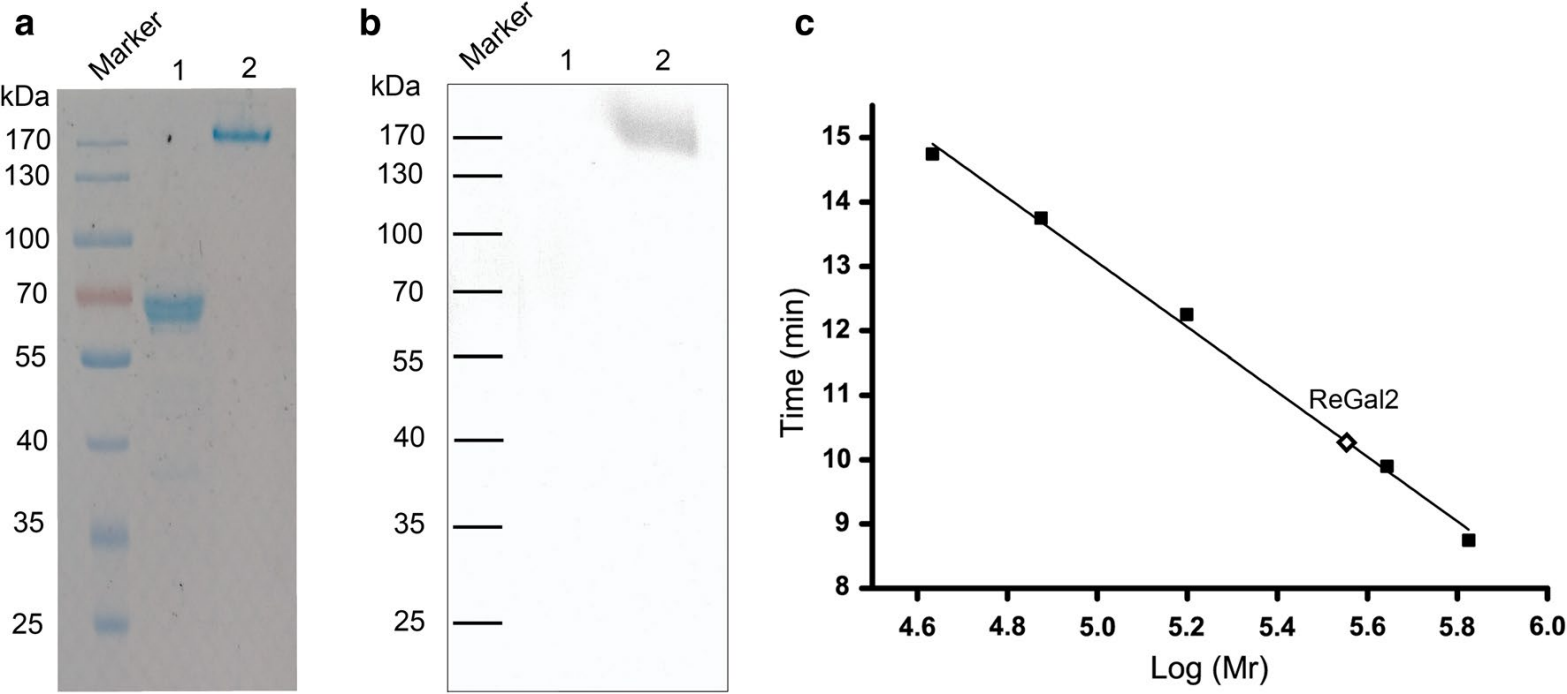
ReGal2 is an oligomer. **a** SDS-PAGE analysis of purified ReGal2 with (lane 1) or without (lane 2) boiling for 15 min. Samples loaded were routinely pretreated with SDS and β-mercaptoethanol. **b** Semi-native PAGE of purified ReGal2 treated with (lane 1) or without (lane 2) 15-min boiling as shown in **a** for determination of its hydrolytic activity toward MUG. **c** Determination of the molecular weight of purified ReGal2 by gel filtration chromatography using a calibration curve plotted with elution volume versus commonlogarithm of protein molecular mass

In most cases, the recombinant ReGal2 migrated on SDS-PAGE as a single, homogenous band with an estimated molecular weight (MW) of 66 kDa, which is a little higher than the predicted MW of a 6 × His-tagged monomer (54 kDa). However, an additional band with a much higher estimated MW, which was verified to be ReGal2 by mass spectrometry, was also observed (Fig. 3e), suggesting that ReGal2 may adopt an oligomeric structure. Further analysis revealed that ReGal2 heat-treated for up to 15 min is present mainly in monomeric forms whereas the untreated enzyme essentially forms the higher MW oligomers (Fig. 4a). Moreover, semi-native PAGE with MUG as the substrate indicated that the active form of ReGal2 is the oligomer but not the monomer (Fig. 4b). The native MW of ReGal2 as determined by gel filtration is 362 kDa (Fig. 4c), suggesting that ReGal2 may be a hexamer.

#### Catalytic properties of ReGal2

**Fig. 5:**
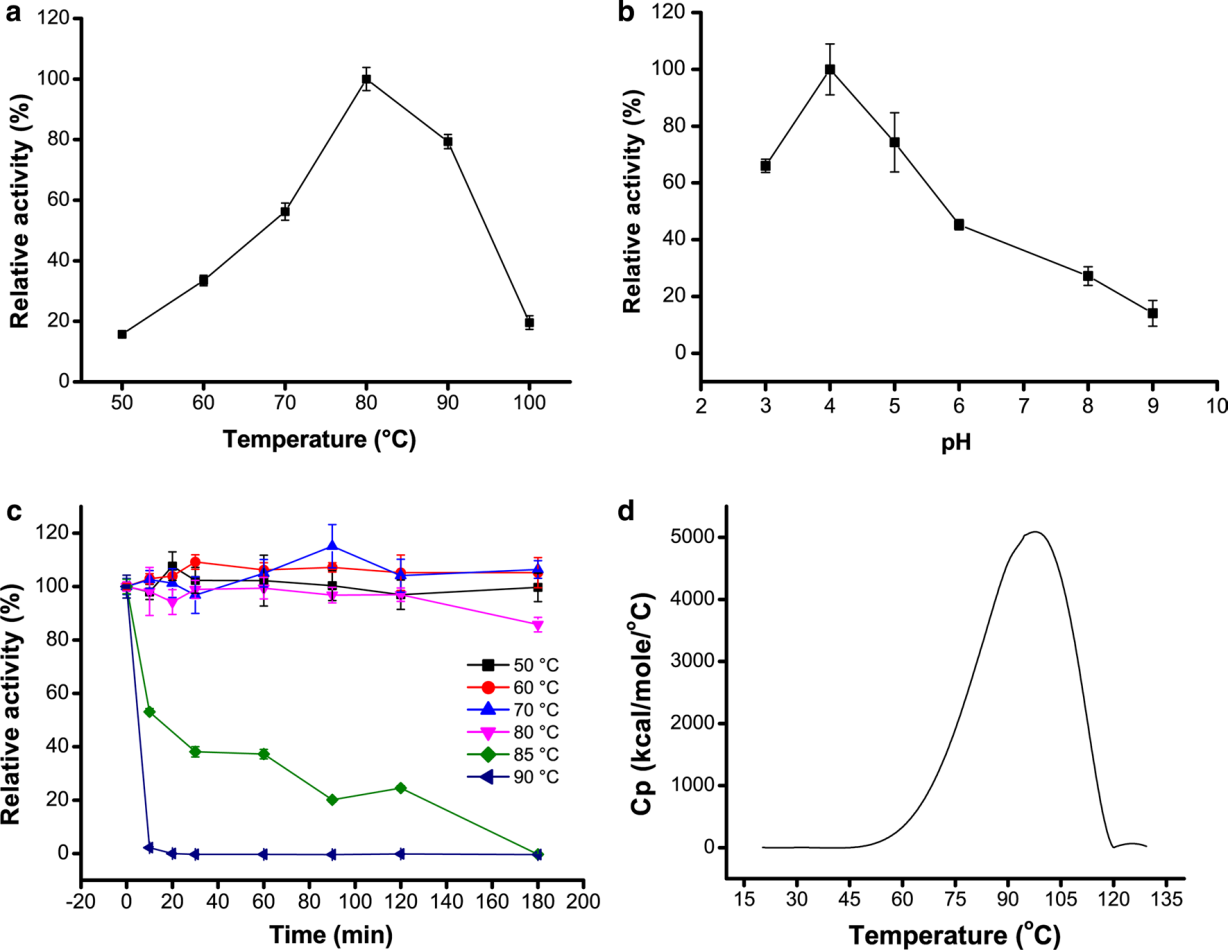
The effects of temperature and pH on the activity of purified ReGal2. **a** Determination of the optimum temperature of ReGal2. **b** Determination of the optimum pH of ReGal2. **c** Determination of the residual activity of ReGal2 after incubation at different temperatures. **d** Thermal unfolding of ReGal2 using DSC analysis

The purified ReGal2 was active over a broad temperature range (50–100 °C) and exhibited an optimum temperature at 80 °C (Fig. 5a). ReGal2 showed the maximum activity at pH 4.0 (Fig. 5b), which is consistent with an acidic optima for most fungal α-galactosidases (optimum pH 4–6) (Table 1). Under the optimum temperature and pH, ReGal2 showed the highest specific activity of 935 U mg^−1^ toward *p*NPGal, which is among the most effiencient fungal α-galactosidases that have been reported (Table 1). Using *p*NPGal as substrate, the *K*_m_, *V*_max_, and k_cat_ values of ReGal2 were determined to be 0.19 ± 0.04 mM, 566.5 ± 15.9 µmol^−1^min^−1^mg^−1^, and 623.1 ± 17.5 s^−1^, respectively. The catalytic efficiency (k_cat_/*K*_m_) of ReGal2 was 3279.7 s^−1^ mM^−1^, which is much higher than the reported thermophilic fungal counterparts, including the first characterized α-galactosidase (ReGal1 in our study) from *R. emersonii* [13].

#### Thermostability of ReGal2

ReGal2 displayed remarkable tolerance to high temperature, retaining 20% of the maximum activity even at 100 °C (Fig. 5a). Further thermal inactivation analyses demonstrated that ReGal2 was 100% active after incubation at 50–70 °C for 3 h (Fig. 5c), and full activity was still maintained after up to 60 h of incubation at 70 °C (data not shown). This is in sharp contrast with the so far characterized thermostable fungal counterparts, which usually lost hydrolytic activities after incubation at 70 °C (Table 1). Of note, ReGal2 retained 80% of its initial activity after incubation of 3 h at 80 °C, whereas *Tt*GalA, one of the most thermophilic α-galactosidase from the thermophilic bacterium *Thermus thermophiles*, retains only 9.7% of its initial activity after the same treatment [31]. Consistently, DSC analysis showed that ReGal2 had a high melting temperature of 97.9 °C (Fig. 5d), an excellent trait that is frequently observed in thermophilic proteins. The above results indicate that ReGal2 is highly thermostable.

#### Effect of metal ions and chemical reagents on the activity of ReGal2

The effect of different metal ions or chemical reagents on the activity of ReGal2 is shown in Tables 3 and 4. Like many other thermophilic fungal counterparts, ReGal2 was remarkably resistant to most metal ions tested, including Ni^2+^, K^+^, Mn^2+^, Cu^2+^, Co^2+^, Zn^2+^, and Mg^2+^, but was sensitive to Ag^+^ (Table 5). ReGal2 also exhibited a relatively high tolerance to NaCl, keeping 76% of its activity with up to 3 M NaCl (Table 6).

**Table 3:** Effect of metal ions on the activities of purified ReGal2

Metal ions	5 mM	10 mM	20 mM
Ni^2+^	105 ± 5.47	104 ± 2.28	98.4 ± 1.6
K^+^	102 ± 2.8	103 ± 8.84	100 ± 1.42
Mn^2+^	114 ± 2.0	108 ± 6.5	104 ± 8.3
Cu^2+^	107 ± 7.3	101 ± 1.3	102 ± 5.9
Co^2+^	107 ± 2.4	105 ± 5.89	101 ± 3.6
Mg^2+^	107 ± 8.1	109 ± 5.06	112 ± 3.7
Zn^2+^	98.95 ± 1.4	105 ± 4.0	95 ± 2.2
Ca^2+^	104 ± 5.6	107 ± 5.2	106 ± 2.8
Ag^+^	0.00 ± 0.16	0.02 ± 0.18	0.94 ± 0.46

**Table 4:** Effect of protein denaturants and EDTA on the activities of purified ReGal2

Compound	0 mM	50 mM	100 mM	200 mM
SDS	100 ± 5.12	106 ± 4.7	103 ± 6.8	104 ± 2.39
Urea	100 ± 0.26	99 ± 2.0	105 ± 2.9	109 ± 1.3
GdnHCl	100 ± 4.3	88 ± 2.6	80 ± 0.8	68 ± 2.3
EDTA	100 ± 5.2	102 ± 4.02	104 ± 2.98	100 ± 5.65
β-mercaptoethanol	100 ± 1.87	96 ± 1.69	104 ± 2.61	106 ± 4.75

**Table 5:** Comparison of the effect of metal ions, protein denaturants, and EDTA on the activity of ReGal2 and several thermophilic counterparts

Metal ions and chemicals	Organisms and references						
	*Talaromyces emersonii* (this study)	*Talaromyces leycettanus* JCM12802 [12]	*Neosartorya fischeri* P1 [10]	*Penicillium canescens* [38]	*Agaricus bisporus* [39]	*Rhizomucor miehei* [32]	*Thermus thermophiles* [31]
Ni^2+^	98.4% (20 mM)	98.5% (5 mM)	98.5% (5 mM)	–	–	90% (2 mM)	–
K^+^	100% (20 mM)	105.9% (5 mM)	77.0% (5 mM)	–	95.3% (10 mM)	102% (2 mM)	–
Mn^2+^	104% (20 mM)	101.2% (5 mM)	103.1% (5 mM)	–	63.9% (10 mM)	95% (2 mM)	20.2% (5 mM)
Cu^2+^	102% (20 mM)	101.1% (5 mM)	93.9% (5 mM)	80% (100 mM)	4.58% (10 mM)	119% (2 mM)	22.2% (5 mM)
Co^2+^	101% (20 mM)	95.6% (5 mM)	95.4% (5 mM)	100% (100 mM)	–	195% (2 mM)	30.1% (5 mM)
Mg^2+^	112% (20 mM)	103.3% (5 mM)	93.6% (5 mM)	–	91.7% (10 mM)	92% (2 mM)	55.6% (5 mM)
Zn^2+^	95% (20 mM)	105.2% (5 mM)	105.0% (5 mM)	100% (100 mM)	95.6% (10 mM)	116% (2 mM)	15.1% (5 mM)
Ca^2+^	106% (20 mM)	98.6% (5 mM)	100.3% (5 mM)	100% (100 mM)	86.9% (10 mM)	129% (2 mM)	27.8% (5 mM)
Ag^+^	0% (5 mM)	0.2% (5 mM)	5.2% (5 mM)	–	0% (10 mM)	4% (2 mM)	–
SDS	104% (200 mM)	0% (5 mM)	19.5% (5 mM)	–	4.53% (50 mM)	5% (2 mM)	1.6% (5 mM)
Urea	109% (200 mM)	–	–	–	–	–	60.0% (5 mM)
GdnHCl	68% (200 mM)	–	–	–	–	–	36.8% (5 mM)
EDTA	100% (200 mM)	103.9% (5 mM)	90.1% (5 mM)	–	106.6% (50 mM)	95% (2 mM)	79.9% (5 mM)
β-Mercaptoethanol	106% (200 mM)	98.6% (5 mM)	77.9% (5 mM)	–	–	–	–

Values represent relative hydrolytic activities of the enzymes in the presence of metal ions or chemicals

**Table 6:** Effect of NaCl on the activities of purified ReGal2

NaCl	0 M	1 M	2 M	3 M
Relative activity (%)	100 ± 3.1	87 ± 1.7	81 ± 3.9	76 ± 0.5

On the other hand, as with other thermophilic counterparts, the enzymatic activity of ReGal2 was hardly affected in the presence of EDTA (Table 5), suggesting that ReGal2 may not have a specific requirement for divalent metal ions as cofactors. Notably, ReGal2 exhibited a significant tolerance to SDS and β-mercaptoethanol with hardly any activity lost when treated with SDS or β-mercaptoethanol up to 200 mM, which is in agreement with the observation that it retained the active oligomeric state during electrophoresis in the presence of these two denaturants. This is quite unique among the so far characterized α-galactosidases, which markedly lost activities in the presence of SDS (Table 5) [9, 10, 12, 31–33]. Even more surprisingly, ReGal2 did not show reduced activity in the presence of urea (up to 200 mM), and maintained approximately up to 80% of its initial activity in the presence of GdnHCl (200 mM), which is a stronger denaturant than urea. This is in sharp contrast with the thermophilic and oligomeric *Tt*GalA, which lost 40% and 64% activities even in the presence of 5 mM urea and GdnHCl, respectively [31]. Together these results indicate that ReGal2 is capable of maintaining its active state even in the presence of potent protein structural denaturants.

Given the many reports indicating that oligomerization stabilizes protein structure, it is reasonably to speculate that oligomerization of ReGal2 contributes to its outstanding tolerance to high temperature and a number of chemicals, especially protein denaturants. Notwithstanding this, exception to this rule does exist wherein two oligomeric thermophilic α-galactosidases, one from *Ganoderma lucidum* and the other from *Thermus thermophilus*, have been reported to be highly sensitive to protein denaturants [31, 34]. We therefore speculated that additional structural features unique to ReGal2 may account for its exceptional structural stability, which merits further investigation.

### Elimination of RFOs from soybean with ReGal2

**Fig. 6:**
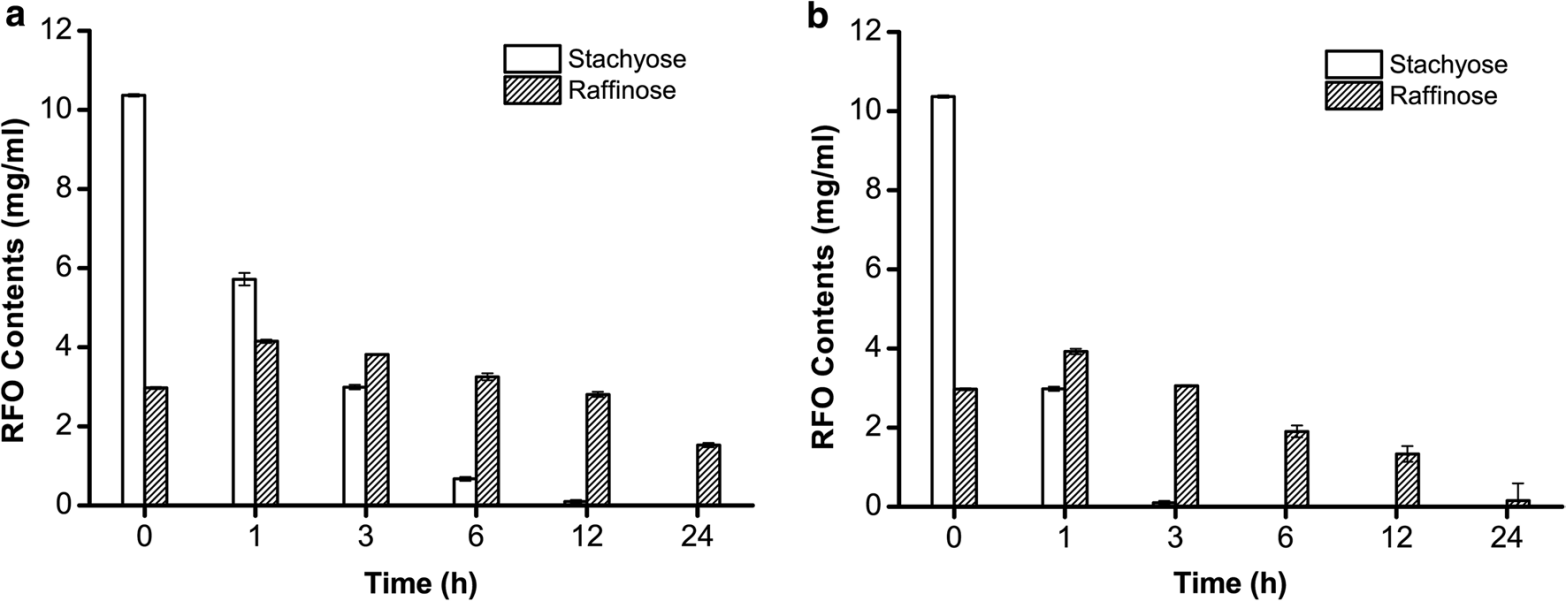
HPLC analyses of hydrolysis products of RFO treated with ReGal2. Reactions were performed at 50 °C (**a**) and 70 °C (**b**), respectively

Soybeans rich in vegetable proteins are used in a variety of food and fodder fields. However, high concentrations of flatulence-causing RFOs have to be removed to improve the soybean utilization efficiency. To test the efficiency of ReGal2 in removing RFOs from soybean materials, we determined changes of stachyose and raffinose content in defatted soybean meal upon treatment with ReGal2. The original amount of stachyose and raffinose in untreated soybean substrate was determined to be 10.37 mg ml^−1^ and 2.96 mg ml^−1^, respectively. A time-course of stachyose and raffinose hydrolysis upon treatment with ReGal2 were analyzed (Fig. 6). Stachyose was found to decrease by 45% and 71% after treatment for 1 h at 50 °C and 70 °C, respectively. The residual stachyose was completely hydrolyzed in 12 h at 50 °C, which was dramatically shortened to 3 h at 70 °C. As a result of stachyose hydrolysis, the raffinose content increased during the early reaction period, but was later on decreased by 55% in 12 h, and completely removed in 24 h after treatment with ReGal2 at 70 °C. These data thus indicate that ReGal2 is able to efficiently remove RFOs from soybeans, and therefore holds great promise in beet sugar processing as well as in food and feed industries for improving the nutritive quality of soybean and other legumes.

## Conclusion

In this study, a thermophilic α-galactosidase encoding gene (*Regal2*) was identified from the thermophilic (hemi)cellulolytic fungus, *R. emersonii*, expressed and purified from an optimized *P. pastoris* expression system with significantly increased production yield. ReGal2 exhibited remarkable thermostability and superior resistance to protein denaturants, *e.g.*, SDS, urea, and GdnHCl. ReGal2 possessed high specific catalytic activity and efficiently removed the anti-nutrient RFOs in soybean. The identified ReGal2 thus holds great potential in relevant biotechnological applications. The optimized *P. pastoris* expression system would also be widely used to increase production yield of heterologous proteins, and therefore contributes to mining more enzymes with desired features.

## Data Availability

All data generated or analyzed during this study are included in this published article.
